# Open-Access Science: A Necessity for Global Public Health

**DOI:** 10.1371/journal.ppat.0010021

**Published:** 2005-10-28

**Authors:** Josefina Coloma, Eva Harris

The world of scientific research and scholarly publishing is undergoing a profound transformation in large part due to the rapid development of information and communication technologies. The Internet and the advent of faster networking capabilities now allow virtually unlimited access to information, remote data gathering, real-time integration of data into databases and models, and online purchasing of research supplies. At the same time, it has opened new possibilities for researchers to communicate with colleagues and with the society in general. However, although some investigators in the developing world are keeping pace with this new reality, the majority are largely excluded from this transformation because of their limited access to scientific information. Particularly relevant to the area of pathogen research, the vast majority of infectious diseases in humans, animals, and plants occurs in the developing world, and efficient communication between local scientists in developing countries and the global community will facilitate advances in knowledge and control of these pathogens. Here we discuss open access and socially responsible philosophies in relation to scientific training, publishing, and intellectual property, and give examples of how we can help keep the developing world fully informed about these new models.

The first step is training partners in developing countries in laboratory techniques and epidemiological skills that complement their existing programs, as well as developing their proficiency in grant proposal writing. A number of organizations have undertaken workshops on such topics around the world, including the World Health Organization (http://www.who.int/en/), the Pan American Health Organization (http://www.paho.org), the Fogarty International Center of the National Institutes of Health (http://www.fic.nih.gov), and the Sustainable Sciences Institute ([SSI]; http://www.ssilink.org), which we represent. SSI is a San Francisco–based organization that helps scientists in underprivileged environments gain access to the resources, technologies, skills, and knowledge they need to conduct cutting-edge research with an impact on public health and to effectively communicate their results [1]. Importantly, these organizations make the content of their courses freely available to participants in the workshops and others, similar to an open-access policy.

Performing research is not the only issue at stake; the scientific community evaluates career advancement and stature primarily by the number and quality of published research articles in peer-reviewed journals, and few investigators in developing countries receive training on how to convert their work into publishable manuscripts. To address this problem, several organizations have begun conducting manuscript-writing workshops in developing countries, including CARE–Centers for Disease Control (http://www.careusa.org/careswork/whatwedo/health/cdc.asp), SSI, and others [1–3]. The objective is to provide the skills needed to transform existing data into publishable material, and increase the likelihood of a manuscript being accepted for publication in a reputable scientific journal. Publications boost investigators' scientific visibility in the international community, help them gain ownership of their research, and improve their chances of competing successfully for external funds. In addition, the findings in published manuscripts have the power to inform and influence local and regional public health policy. SSI has conducted four manuscript-writing workshops throughout Latin America, and the enthusiastic response indicates that this is indeed fulfilling an unmet need.

Dissemination of research is an integral part of the scientific process, and scientific journals are the classical channel used to achieve this. Medical societies, universities, research institutions, and libraries in the developed world have traditionally covered the costs of most journal subscriptions, shielding individual scientists from the steep increases in subscription prices. For example, subscriptions to scientific journals are reported to have increased 227% between 1986 and 2002 (see Graph 2 and Table 2 in [4]). However, scientists in developed countries have remained largely oblivious to this financial threat to scholarship and indifferent to the costs because they have enthusiastically embraced the desktop accessibility to journals.

In the developing world, scientists face a greater challenge to remain informed about the progress in their fields of research. Although they are disproportionately affected by infectious diseases, they are excluded from the relevant information that might help them cure, control, and manage the effects of these diseases. Institutions and libraries have been unable to keep up with increases in journal pricing, so individual researchers have had to cover the costs of the few journals to which they subscribe; however, most researchers simply cannot afford to do this and remain excluded from access to information and from the possibility of publishing in a widely distributed journal. In the last few years, initiatives such as the Health InterNetwork Access to Research Initiative (http://www.who.int/hinari/en, http://www.healthinternetwork.net) have made access to online journals, although not always current, a possibility for those researchers with internet access—constituting a large step toward reducing the medical and scientific information gap between rich and poor countries. Nonetheless, the digital divide is still great in many regions. Latin America, for instance, is a region with islands of progress, where only a few privileged researchers have access to state-of-the-art scientific and technological infrastructures. Most national research and education networks are based on low-speed commercial internet services; there is limited global connectivity and great disparity in the levels of development within and between countries. As a result, the region as a whole is not positioned to participate effectively in global research.

Although many of us base our research on crucial collaborations with researchers in developing countries, we often do not fully understand the limitations they face every day when they try to access information or communicate their findings. As a result, it is often difficult for them to compete at the same level with counterparts in a privileged environment. We have failed to support our colleagues abroad, ignoring their isolation because we are encouraged to publish our research in high-profile journals, which will increase our competitiveness and access to further funding. This individualistic and self-perpetuating cycle can only be broken if researchers and funding agencies alike shift their mindset. The first principle we should all support is that publicly funded research should be made publicly available through the most appropriate open-access channel. Funding agencies and reviewers need to give researchers credit, not penalize them, for efforts to publish in new open-access media. Even with strict peer review, new electronic journals will not immediately attain the same impact status as traditional print journals, but they will have a greater reach and a larger global influence. In fact, it has been shown that online accessibility increases the citation rate and, thus, the impact of a journal by 157% [5]. This new online publishing venue might be the only way that scientists in the developing world conducting highly relevant research can make their data available to the world. It might also be the only means for them to obtain the most recent and relevant information for their research.

Recent developments in Europe and the United States led by the European Science Foundation (Open Archives Initiative, http://www.openarchives.org), the Public Library of Science (http://www.plos.org), and PubMed Central (http://www.pubmedcentral.nih.gov) have focused on an open-access initiative that attempts to establish a norm of no-cost dissemination of author-copyrighted articles. Since 1999, the Open Archives Initiative has developed and promoted standards to facilitate the dissemination of content, enhanced access to electronic print archives to promote the concept of electronic preprint, and created generic mechanisms to harvest data from repositories. Fortunately, there are numerous examples of systems that provide software allowing access to the research literature online through author-driven and institutional archiving, helping construct a global digital library of scientific information.

Finally, global access to scientific knowledge and the fruits of that knowledge also extend to the products that result from scientific endeavors. This, necessarily, treads into the hotly contested area of intellectual property. But, in fact, win–win solutions exist that allow profits to be generated from inventions and at the same time ensure that these products are available “at cost” in developing countries. For example, a new trend of licensing models is under way at the University of California (UC) Berkeley, where several innovations with practical use have been subjects of this novel initiative. SSI helped set the stage for this concept by developing an agreement with the UC Berkeley Office of Technology Licensing to obtain the right to market one of UC Berkeley's patents “at cost” in the developing world. This was swiftly followed by several widely publicized agreements involving royalty-free licenses for developing countries' use of products—including the Gates-funded project for artemisinin synthesis in *Escherichia coli* being conducted at UC Berkeley, the Institute for OneWorld Health, and Amyris Biotechnologies, and an agreement between UC Berkeley and Samoa for development of plant-derived pharmaceuticals. Importantly, a new movement for “socially responsible licensing” has been born, led by UC Berkeley, Harvard University, Massachusetts Institute of Technology, Yale University, University of Minnesota, and Columbia University, to share ideas on bringing access to technology and medical treatments to the developing world.

We believe the whole spectrum of scientific endeavor should be as open access as possible, from training in laboratory and epidemiological techniques, proposal writing, and manuscript-writing skills to open-access publishing and socially responsible intellectual property policies. In this way, a new door of opportunity can be opened so that the fruits of our scientific breakthroughs are disseminated worldwide and benefit global public health. 

## 

**Figure 1:**
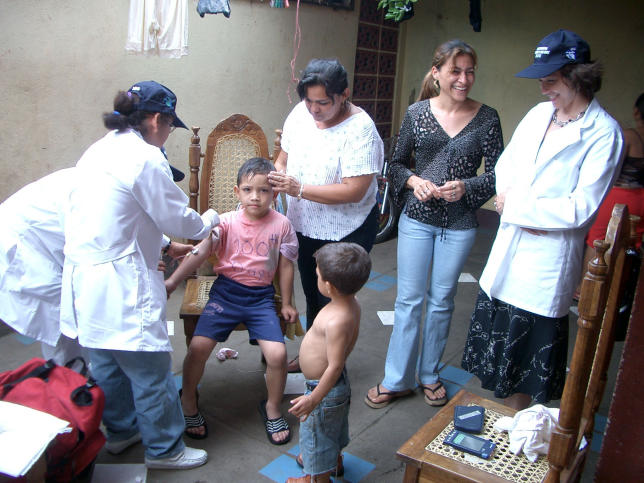
Annual Collection of Serological Samples, Managua, Nicaragua, 2005 The collection of samples, monitored here by Eva Harris (right), is part of the UC Berkeley/SSI pediatric dengue cohort study in Managua, Nicaragua. Objectives of this study include investigating the natural history and risk factors for dengue virus transmission and disease, obtaining biological samples in parallel with clinical and epidemiological information, and establishing a site for a future phase III trial of a safe tetravalent dengue vaccine. (Photo: Alejandro Belli)

**Figure 2:**
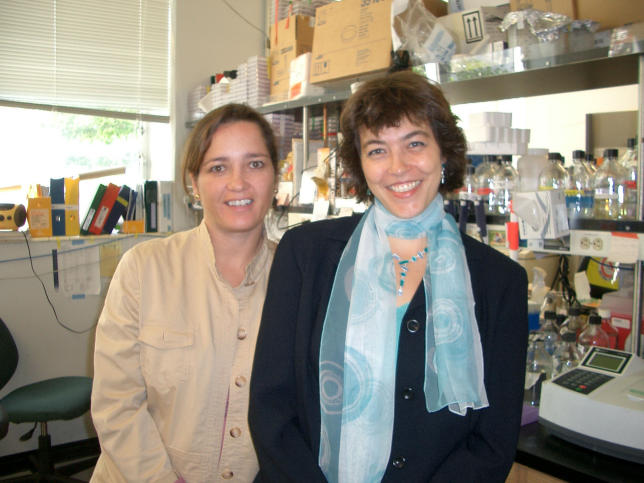
Josefina Coloma (left) and Eva Harris (right) in their laboratory at the School of Public Health, UC Berkeley (Photo: Jennifer Kyle)

## Abbreviations

SSI: Sustainable Sciences Institute
UC: University of California

## References

[ppat-0010021-b01] Harris E (2004). Scientific capacity building in developing countries. EMBO Rep.

[ppat-0010021-b02] Glew RH (2002). The true meaning of technology transfer. Electron J Biotechnol 5.

[ppat-0010021-b03] Churchill RE (2002). Preventing data drain: An international project. Sci Ed.

[ppat-0010021-b04] Kyrillidou M, Young M (2005). ARL statistics 2001–2002.

[ppat-0010021-b05] Lawrence S (2001). Free online availability substantially increases a paper's impact.

